# Poly[diaqua­(μ_4_-2,5-dicarb­oxy­benzene-1,4-dicarboxyl­ato-κ^4^ *O* ^1^:*O* ^2^:*O* ^4^:*O* ^5^)(μ_2_-2,5-dicarb­oxy­benzene-1,4-dicarboxyl­ato-κ^2^ *O* ^1^:*O* ^4^)bis­(1,10-phenanthroline-κ^2^ *N*,*N*′)dimanganese(II)]

**DOI:** 10.1107/S1600536812035441

**Published:** 2012-08-23

**Authors:** Kai-Long Zhong

**Affiliations:** a Department of Applied Chemistry, Nanjing College of Chemical Technology, Nanjing 210048, People’s Republic of China

## Abstract

In the title compound, [Mn_2_(C_10_H_4_O_8_)_2_(C_12_H_8_N_2_)_2_(H_2_O)_2_]_*n*_, the Mn^2+^ ion has a slightly distorted octa­hedral N_2_O_4_ coordination geometry being coordinated by one aqua O atom, two N atoms of the chelating 1,10-phenanthroline ligand and three carboxyl O atoms from three 2,5-dicarb­oxy­benzene-1,4-dicarboxyl­ate (H_2_btec^2−^) ligands. The H_2_btec^2−^ anion exhibits two different coordination modes, *viz*. μ_2_ and μ_4_. Both of the H_2_btec^2−^ anions are located on special positions (inversion centers). The μ_4_-anion bridges adjacent Mn^II^ atoms, forming a chain along the *a* axis. Adjacent chains are further bridged by μ_2_-anions, resulting in a two-dimensional layered polymer parallel to (011). In the crystal, extensive carb­oxy–carboxyl­ate O—H⋯O and water–carboxyl­ate O—H⋯O inter­actions lead to the formation of a three-dimensional supra­molecular network.

## Related literature

For isotypic structures, see: Hu *et al.* (2004 ▶); Yu *et al.* (2007 ▶). For background to manganese complexes and phenanthroline complexes, see: Zhu *et al.* (2006 ▶); Zhong *et al.* (2009 ▶); Cui *et al.* (2010 ▶); Zhong (2011 ▶). For background to coordination polymers, see: Batten & Robson (1998 ▶); Fabelo *et al.* (2008 ▶); Liu *et al.* (2007 ▶); Li *et al.* (2003 ▶); Zhang *et al.* (2010 ▶).
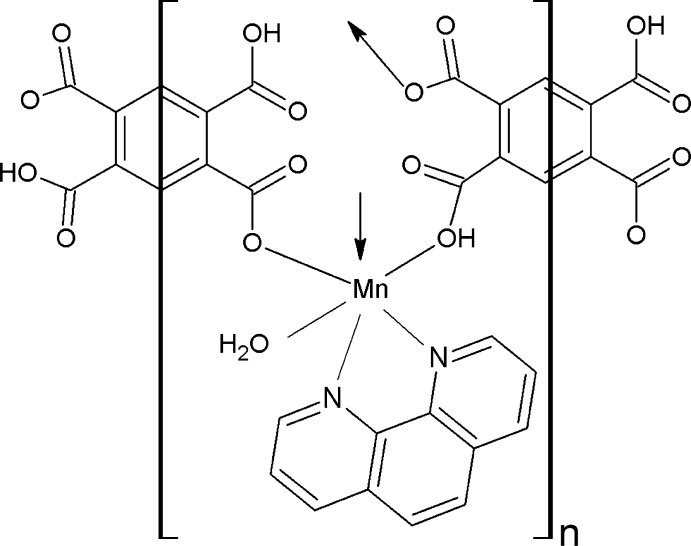


## Experimental

### 

#### Crystal data

[Mn_2_(C_10_H_4_O_8_)_2_(C_12_H_8_N_2_)_2_(H_2_O)_2_]
*M* *_r_* = 1010.58
Triclinic, 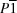

*a* = 9.880 (2) Å
*b* = 10.246 (2) Å
*c* = 11.272 (2) Å
α = 86.29 (3)°
β = 71.82 (3)°
γ = 65.32 (3)°
*V* = 982.2 (5) Å^3^
*Z* = 1
Mo *K*α radiation
μ = 0.74 mm^−1^
*T* = 223 K
0.50 × 0.50 × 0.30 mm

#### Data collection

Rigaku Mercury CCD diffractometer
Absorption correction: multi-scan (*REQAB*: Jacobson, 1998 ▶) *T*_min_ = 0.710, *T*_max_ = 0.810
9376 measured reflections
4403 independent reflections
3757 reflections with *I* > 2σ(*I*)
*R*_int_ = 0.025

#### Refinement

*R*[*F* ^2^ > 2σ(*F* ^2^)] = 0.031
*wR*(*F* ^2^) = 0.081
*S* = 1.05
4403 reflections
315 parameters
H atoms treated by a mixture of independent and constrained refinement
Δρ_max_ = 0.42 e Å^−3^
Δρ_min_ = −0.45 e Å^−3^

### 

Data collection: *CrystalClear* (Rigaku, 2007 ▶); cell refinement: *CrystalClear*; data reduction: *CrystalClear*; program(s) used to solve structure: *SHELXS97* (Sheldrick, 2008 ▶); program(s) used to refine structure: *SHELXL97* (Sheldrick, 2008 ▶); molecular graphics: *XP* in *SHELXTL* (Sheldrick, 2008 ▶); software used to prepare material for publication: *SHELXTL*.

## Supplementary Material

Crystal structure: contains datablock(s) global, I. DOI: 10.1107/S1600536812035441/zq2172sup1.cif

Structure factors: contains datablock(s) I. DOI: 10.1107/S1600536812035441/zq2172Isup2.hkl

Additional supplementary materials:  crystallographic information; 3D view; checkCIF report

## Figures and Tables

**Table 1:** Selected bond lengths (Å)

Mn1—O1*W*	2.1576 (17)
Mn1—O5^i^	2.1830 (12)
Mn1—O7	2.1852 (13)
Mn1—O1	2.2001 (12)
Mn1—N2	2.2314 (14)
Mn1—N1	2.2317 (17)

Symmetry code: (i) 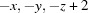.

**Table 2:** Hydrogen-bond geometry (Å, °)

*D*—H⋯*A*	*D*—H	H⋯*A*	*D*⋯*A*	*D*—H⋯*A*
O8—H8⋯O1	0.82	1.75	2.5176 (16)	155
O4—H4⋯O6^ii^	0.82	1.81	2.592 (2)	158
O1*W*—H1*WB*⋯O6^i^	0.79 (2)	1.95 (3)	2.7108 (19)	160 (2)
O1*W*—H1*WA*⋯O2^iii^	0.80 (2)	1.94 (2)	2.7117 (18)	162 (2)

Symmetry codes: (i) 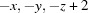; (ii) 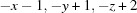; (iii) 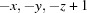.

**Fig. 1.**
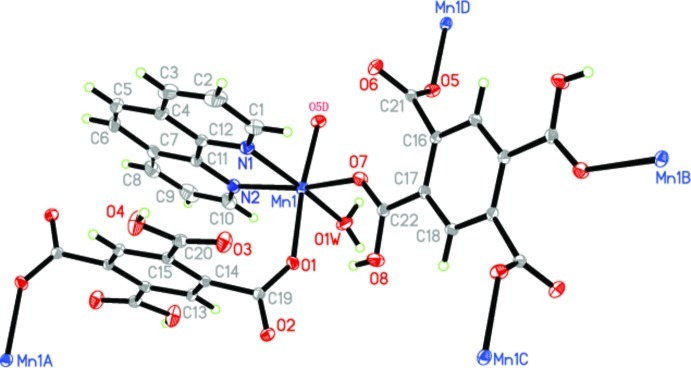
The asymmetric unit of the title compound (displacement ellipsoids drawn at the 35% probability level). Unlabeled atoms are related to the labelled atoms by symmetry operators (symmetry codes: A = -x, 1 - y, 1 - z; B = 1 - x, -y, 2 - z; C = x - 1, y, z; D = -x, -y, 2 - z).

**Fig. 2.**
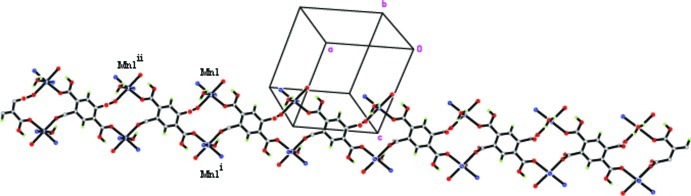
The one-dimensional chain structure of the title compound. Dashed lines indicate hydrogen bonds. All C atoms of the phenantroline and µ2-H2btec ligands have been omitted for clarity (symmetry codes: i = 1 + x, y, z; ii = -x, -y, 2 - z).

**Fig. 3.**
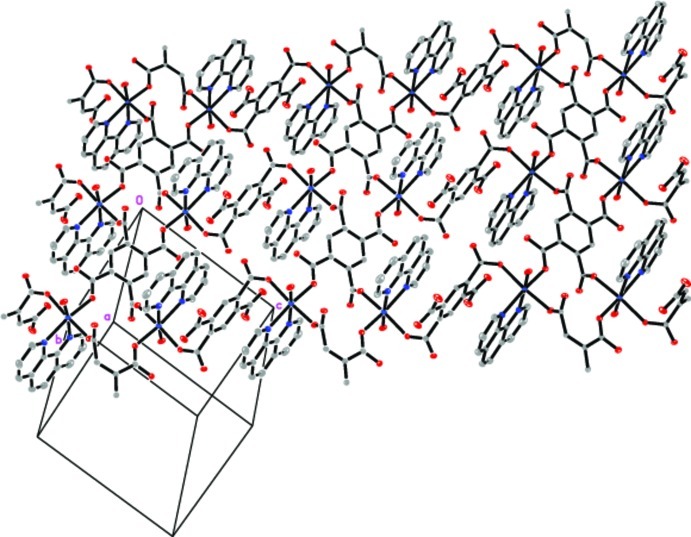
The two-dimensional layer structure of the title compound viewed along the a axis. All H atoms have been omitted for clarity.

**Fig. 4.**
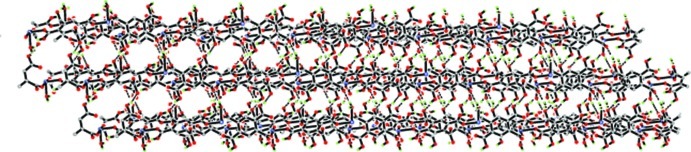
Hydrogen-bonding interactions between adjacent layers of the title compound. Dashed lines indicate hydrogen bonds.

## supplementary crystallographic information

### Comment

Recently, the self-assembly of coordination polymers and the crystal engineering of metal-organic coordination frameworks have received much attention (Batten & Robson, 1998; Liu *et al.*, 2007; Zhang *et al.*, 2010). The 1,2,4,5-benzenetetracarboxylate acid and 1,10-phenanthroline (phen) have also been widely employed as polydentate ligands in coordination reactions and in the construction of supermolecular networks (Li *et al.*, 2003; Fabelo *et al.*, 2008). In the past few years, we have synthesized and reported many metal-Phen complexes such as manganese (Zhu *et al.*, 2006), nickel (Zhong *et al.*, 2009), zinc (Cui *et al.*, 2010) and cadmium (Zhong, 2011) complexes . The title compound [Mn(phen)(µ_2_ –H_2_btec)_1/2_(µ_4_– H_2_btec)_1/2_(H_2_O)], (I), was obtained during an attempt to synthesize a mixed-ligand complex of Mn^II^ with *phen* and 1,2,4,5-benzenetetracarboxylate ligand *via* a hydrothermal reaction.

The title compound is isomorphic with previously reported cobalt(II) and zinc(II) analogs (Hu *et al.*, 2004; Yu *et al.*, 2007). The X-ray diffraction study indicates that the Mn^II^ centers exhibit a slightly distorted octahedral MnN_2_O_4_ coordination environment. Atom Mn1 is coordinated by one aqua O atom (O1W), two N atoms (N1 and N2) from a bridging 1,10-phenanthroline ligand and three carboxyl O atoms (O1, O7 and O5D) from three H_2_btec^2-^ anions. The N1, N2, O1W and O7 atoms occupy the equatorial sites, while O1 and O5D occupy the axial positions (Fig. 1 and Table 1). The Mn—N1 and Mn—N2 bond distances are 2.2317 (17) Å and 2.2314 (14) Å, respectively, and the N1—Ni—N2 bite angle is 75.32 (6)°. The bond lengths of Mn—O range from 2.1576 (17) Å to 2.2001 (12) Å. The H_2_btec^2-^ anion exhibits two different coordination modes, µ_2_-H_2_btec^2-^ and µ_4_-H_2_btec^2-^. The mean planes defined by µ_2_-H_2_btec^2-^ and its neighbour phenantroline ring are almost parallel [the dihedral angle is 5.18 (4)°], while the mean planes defined by µ_4_-H_2_btec^2-^ and its neighbour phenantroline ring are oriented at 64.33 (4)°, those are in agreement with that observed in [Co(C_10_H_4_O_8_) (C_12_H_8_N_2_)(H_2_O)] (Hu *et al.*, 2004). The µ_4_-H_2_btec^2-^ anions link neighbour Mn^II^ cations, giving rise to one-dimensional chains running along the *a* axis. The distances between two neighbour Mn atoms are 9.880 (2) Å [Mn1—Mn1 (1 + *x*, *y*, *z*)] and 5.880 (2) Å [Mn1—Mn1 (-*x*, -*y*, 2 - *z*)], respectively (Fig. 2). The adjacent one-dimensional chains are further crosslinked by bridging µ_2_-H_2_btec^2-^ anions, leading to a two-dimensional layered polymer (Fig. 3). Intermolecular O8—H8···O1 hydrogen bond and π-π interaction between the parallel phenantroline and µ_2_-H_2_btec^2-^ ligands help to further stabilize the layered structure (Fig.3 and Table 2). In the crystal structure, the two-dimensional polymeric layers are linked by carboxylic acid O—H···O carboxyl and water O—H···O carboxyl hydrogen bonds to form a three-dimensional supramolecular network structure (Fig.4).

### Experimental

0.1 mmol MnSO_4_^.^2H_2_O, 0.1 mmol phen, 0.1 mmol 1,2,4,5-benzenetetracarboxylic acid and 3.0 ml water were mixed and placed in a thick Pyrex tube, which was sealed and heated to 383 K for 72 h, whereupon pale-yellow block-shaped crystals were obtained.

### Refinement

All non-hydrogen atoms were refined anisotropically. The H atoms were positioned geometrically and allowed to ride on their parent atoms, with C—H = 0.93 Å, O—H = 0.82 Å, *U*_iso_(H) = 1.2*U*_eq_(C) and *U*_iso_(H) = 1.5*U*_eq_(O). The H atoms of the water molecules were located in difference Fourier maps and freely refined.

### Crystal data

**Table:**

[Mn_2_(C_10_H_4_O_8_)_2_(C_12_H_8_N_2_)_2_(H_2_O)_2_]	*Z* = 1
*M**_r_* = 1010.58	*F*(000) = 514
Triclinic, *P*1	*D*_x_ = 1.708 Mg m^−^^3^
Hall symbol: -P 1	Mo *K*α radiation, λ = 0.71073 Å
*a* = 9.880 (2) Å	Cell parameters from 4733 reflections
*b* = 10.246 (2) Å	θ = 3.5–27.5°
*c* = 11.272 (2) Å	µ = 0.74 mm^−^^1^
α = 86.29 (3)°	*T* = 223 K
β = 71.82 (3)°	Prism, pale yellow
γ = 65.32 (3)°	0.50 × 0.50 × 0.30 mm
*V* = 982.2 (5) Å^3^	

### Data collection

**Table:**

Rigaku Mercury CCD diffractometer	4403 independent reflections
Radiation source: fine-focus sealed tube	3757 reflections with *I* > 2σ(*I*)
Graphite Monochromator monochromator	*R*_int_ = 0.025
Detector resolution: 28.5714 pixels mm^-1^	θ_max_ = 27.5°, θ_min_ = 3.5°
ω scans	*h* = −11→12
Absorption correction: multi-scan (*REQAB*: Jacobson, 1998)	*k* = −12→13
*T*_min_ = 0.710, *T*_max_ = 0.810	*l* = −13→14
9376 measured reflections	

### Refinement

**Table:**

Refinement on *F*^2^	Primary atom site location: structure-invariant direct methods
Least-squares matrix: full	Secondary atom site location: difference Fourier map
*R*[*F*^2^ > 2σ(*F*^2^)] = 0.031	Hydrogen site location: inferred from neighbouring sites
*wR*(*F*^2^) = 0.081	H atoms treated by a mixture of independent and constrained refinement
*S* = 1.05	*w* = 1/[σ^2^(*F*_o_^2^) + (0.0441*P*)^2^] where *P* = (*F*_o_^2^ + 2*F*_c_^2^)/3
4403 reflections	(Δ/σ)_max_ < 0.001
315 parameters	Δρ_max_ = 0.42 e Å^−^^3^
0 restraints	Δρ_min_ = −0.45 e Å^−^^3^

### Special details

**Table:**

Geometry. All e.s.d.'s (except the e.s.d. in the dihedral angle between two l.s. planes) are estimated using the full covariance matrix. The cell e.s.d.'s are taken into account individually in the estimation of e.s.d.'s in distances, angles and torsion angles; correlations between e.s.d.'s in cell parameters are only used when they are defined by crystal symmetry. An approximate (isotropic) treatment of cell e.s.d.'s is used for estimating e.s.d.'s involving l.s. planes.
Refinement. Refinement of *F*^2^ against ALL reflections. The weighted *R*-factor *wR* and goodness of fit *S* are based on *F*^2^, conventional *R*-factors *R* are based on *F*, with *F* set to zero for negative *F*^2^. The threshold expression of *F*^2^ > σ(*F*^2^) is used only for calculating *R*-factors(gt) *etc*. and is not relevant to the choice of reflections for refinement. *R*-factors based on *F*^2^ are statistically about twice as large as those based on *F*, and *R*- factors based on ALL data will be even larger.

### Fractional atomic coordinates and isotropic or equivalent isotropic displacement parameters (Å^2^)

**Table:**

	*x*	*y*	*z*	*U*_iso_*/*U*_eq_	
Mn1	0.03585 (3)	0.13877 (2)	0.76264 (2)	0.01653 (9)	
O1	−0.07365 (14)	0.18231 (12)	0.61328 (10)	0.0203 (2)	
O1W	0.19967 (17)	−0.06467 (13)	0.65855 (13)	0.0287 (3)	
H1WB	0.235 (3)	−0.112 (2)	0.709 (2)	0.048 (7)*	
H1WA	0.191 (3)	−0.113 (2)	0.610 (2)	0.051 (7)*	
O2	−0.20089 (15)	0.27574 (12)	0.47561 (11)	0.0251 (3)	
O3	−0.36828 (15)	0.48967 (13)	0.70890 (11)	0.0289 (3)	
O4	−0.38216 (15)	0.71264 (12)	0.71302 (12)	0.0344 (3)	
H4	−0.4697	0.7305	0.7625	0.052*	
O5	−0.16064 (13)	−0.06578 (11)	1.09959 (10)	0.0196 (2)	
O6	−0.32453 (13)	0.16588 (11)	1.15378 (10)	0.0201 (2)	
O7	−0.15899 (14)	0.08266 (12)	0.86097 (10)	0.0230 (3)	
O8	−0.24706 (16)	0.05474 (14)	0.70863 (10)	0.0302 (3)	
H8	−0.1745	0.0730	0.6647	0.045*	
N1	−0.10378 (17)	0.35804 (14)	0.86211 (12)	0.0201 (3)	
N2	0.19008 (17)	0.25423 (14)	0.69255 (12)	0.0200 (3)	
C1	−0.2460 (2)	0.40702 (19)	0.94468 (16)	0.0277 (4)	
H1A	−0.2977	0.3468	0.9626	0.033*	
C2	−0.3227 (3)	0.5448 (2)	1.00651 (18)	0.0380 (5)	
H2A	−0.4231	0.5759	1.0636	0.046*	
C3	−0.2453 (3)	0.63274 (19)	0.98044 (18)	0.0389 (5)	
H3A	−0.2940	0.7250	1.0199	0.047*	
C4	−0.0943 (2)	0.58473 (18)	0.89512 (16)	0.0298 (4)	
C5	−0.0026 (3)	0.6674 (2)	0.86821 (19)	0.0396 (5)	
H5A	−0.0461	0.7597	0.9068	0.048*	
C6	0.1431 (3)	0.6146 (2)	0.78925 (19)	0.0394 (5)	
H6A	0.1996	0.6706	0.7747	0.047*	
C7	0.2162 (2)	0.4723 (2)	0.72534 (16)	0.0298 (4)	
C8	0.3693 (3)	0.4120 (2)	0.64430 (18)	0.0376 (5)	
H8A	0.4301	0.4641	0.6276	0.045*	
C9	0.4304 (2)	0.2758 (2)	0.58920 (19)	0.0386 (5)	
H9A	0.5329	0.2340	0.5354	0.046*	
C10	0.3351 (2)	0.2004 (2)	0.61544 (17)	0.0289 (4)	
H10A	0.3762	0.1085	0.5768	0.035*	
C11	0.1296 (2)	0.38922 (17)	0.74896 (15)	0.0211 (4)	
C12	−0.0266 (2)	0.44510 (17)	0.83610 (15)	0.0212 (4)	
C13	0.08667 (19)	0.36364 (16)	0.44035 (14)	0.0169 (3)	
H13A	0.1458	0.2719	0.3997	0.020*	
C14	−0.06214 (19)	0.39624 (16)	0.52328 (13)	0.0152 (3)	
C15	−0.15027 (19)	0.53453 (16)	0.58379 (14)	0.0161 (3)	
C16	−0.39312 (18)	0.02498 (15)	1.04387 (13)	0.0144 (3)	
C17	−0.37723 (18)	0.02458 (15)	0.91572 (14)	0.0149 (3)	
C18	−0.48391 (18)	−0.00020 (15)	0.87421 (13)	0.0156 (3)	
H16A	−0.4723	−0.0001	0.7891	0.019*	
C19	−0.12121 (19)	0.27874 (16)	0.53878 (13)	0.0159 (3)	
C20	−0.3108 (2)	0.57403 (17)	0.67429 (14)	0.0190 (3)	
C21	−0.28096 (18)	0.04445 (16)	1.10044 (13)	0.0149 (3)	
C22	−0.25052 (19)	0.05606 (16)	0.82447 (14)	0.0168 (3)	

### Atomic displacement parameters (Å^2^)

**Table:**

	*U*^11^	*U*^22^	*U*^33^	*U*^12^	*U*^13^	*U*^23^
Mn1	0.01545 (15)	0.01648 (14)	0.02046 (14)	−0.00844 (11)	−0.00689 (10)	0.00152 (9)
O1	0.0245 (7)	0.0232 (6)	0.0220 (6)	−0.0159 (5)	−0.0120 (5)	0.0094 (5)
O1W	0.0373 (9)	0.0203 (6)	0.0300 (7)	−0.0062 (6)	−0.0199 (6)	−0.0032 (6)
O2	0.0358 (8)	0.0259 (6)	0.0276 (6)	−0.0192 (6)	−0.0205 (6)	0.0070 (5)
O3	0.0233 (7)	0.0290 (6)	0.0343 (7)	−0.0167 (6)	0.0000 (6)	−0.0006 (5)
O4	0.0216 (7)	0.0234 (6)	0.0446 (8)	−0.0094 (6)	0.0095 (6)	−0.0094 (6)
O5	0.0162 (6)	0.0204 (6)	0.0216 (6)	−0.0043 (5)	−0.0095 (5)	−0.0006 (4)
O6	0.0176 (6)	0.0187 (6)	0.0255 (6)	−0.0074 (5)	−0.0079 (5)	−0.0029 (5)
O7	0.0228 (7)	0.0365 (7)	0.0204 (6)	−0.0216 (6)	−0.0092 (5)	0.0078 (5)
O8	0.0372 (8)	0.0556 (8)	0.0172 (6)	−0.0376 (7)	−0.0102 (5)	0.0109 (5)
N1	0.0202 (8)	0.0203 (7)	0.0195 (7)	−0.0068 (6)	−0.0082 (6)	0.0016 (5)
N2	0.0200 (8)	0.0213 (7)	0.0218 (7)	−0.0109 (6)	−0.0079 (6)	0.0041 (5)
C1	0.0222 (10)	0.0311 (9)	0.0241 (8)	−0.0055 (8)	−0.0072 (7)	−0.0014 (7)
C2	0.0318 (12)	0.0358 (11)	0.0286 (10)	0.0032 (9)	−0.0086 (9)	−0.0069 (8)
C3	0.0505 (14)	0.0212 (9)	0.0299 (10)	0.0043 (9)	−0.0186 (10)	−0.0056 (8)
C4	0.0493 (13)	0.0180 (8)	0.0249 (9)	−0.0098 (8)	−0.0217 (9)	0.0030 (7)
C5	0.0750 (18)	0.0220 (9)	0.0355 (11)	−0.0236 (11)	−0.0320 (12)	0.0078 (8)
C6	0.0758 (18)	0.0343 (10)	0.0394 (11)	−0.0414 (12)	−0.0366 (12)	0.0188 (9)
C7	0.0478 (13)	0.0364 (10)	0.0284 (9)	−0.0322 (10)	−0.0249 (9)	0.0169 (8)
C8	0.0450 (13)	0.0556 (13)	0.0389 (11)	−0.0404 (11)	−0.0245 (10)	0.0220 (10)
C9	0.0246 (11)	0.0605 (14)	0.0360 (10)	−0.0250 (10)	−0.0091 (9)	0.0158 (10)
C10	0.0230 (10)	0.0335 (10)	0.0291 (9)	−0.0128 (8)	−0.0063 (8)	0.0066 (8)
C11	0.0299 (10)	0.0228 (8)	0.0201 (8)	−0.0158 (8)	−0.0149 (7)	0.0074 (6)
C12	0.0304 (10)	0.0165 (8)	0.0198 (8)	−0.0084 (7)	−0.0145 (7)	0.0038 (6)
C13	0.0190 (9)	0.0149 (7)	0.0179 (7)	−0.0075 (6)	−0.0064 (6)	0.0006 (6)
C14	0.0179 (9)	0.0176 (7)	0.0146 (7)	−0.0097 (6)	−0.0082 (6)	0.0040 (6)
C15	0.0163 (8)	0.0184 (8)	0.0150 (7)	−0.0076 (6)	−0.0063 (6)	0.0024 (6)
C16	0.0143 (8)	0.0121 (7)	0.0181 (7)	−0.0055 (6)	−0.0067 (6)	0.0007 (6)
C17	0.0139 (8)	0.0144 (7)	0.0172 (7)	−0.0066 (6)	−0.0049 (6)	0.0022 (6)
C18	0.0167 (9)	0.0168 (7)	0.0142 (7)	−0.0073 (6)	−0.0057 (6)	0.0021 (6)
C19	0.0159 (9)	0.0166 (7)	0.0137 (7)	−0.0076 (6)	−0.0015 (6)	−0.0004 (6)
C20	0.0175 (9)	0.0222 (8)	0.0182 (7)	−0.0087 (7)	−0.0061 (7)	0.0003 (6)
C21	0.0147 (8)	0.0193 (8)	0.0133 (7)	−0.0101 (7)	−0.0038 (6)	0.0025 (6)
C22	0.0166 (9)	0.0171 (7)	0.0186 (7)	−0.0087 (7)	−0.0061 (6)	0.0033 (6)

### Geometric parameters (Å, º)

**Table:**

Mn1—O1W	2.1576 (17)	C4—C12	1.408 (2)
Mn1—O5^i^	2.1830 (12)	C4—C5	1.437 (3)
Mn1—O7	2.1852 (13)	C5—C6	1.331 (3)
Mn1—O1	2.2001 (12)	C5—H5A	0.9300
Mn1—N2	2.2314 (14)	C6—C7	1.448 (3)
Mn1—N1	2.2317 (17)	C6—H6A	0.9300
O1—C19	1.2858 (19)	C7—C8	1.391 (3)
O1W—H1WB	0.79 (2)	C7—C11	1.402 (2)
O1W—H1WA	0.80 (2)	C8—C9	1.368 (3)
O2—C19	1.2250 (19)	C8—H8A	0.9300
O3—C20	1.2040 (19)	C9—C10	1.405 (3)
O4—C20	1.325 (2)	C9—H9A	0.9300
O4—H4	0.8200	C10—H10A	0.9300
O5—C21	1.250 (2)	C11—C12	1.437 (3)
O5—Mn1^i^	2.1830 (12)	C13—C14	1.385 (2)
O6—C21	1.2581 (18)	C13—C15^ii^	1.400 (2)
O7—C22	1.2304 (19)	C13—H13A	0.9300
O8—C22	1.2963 (18)	C14—C15	1.398 (2)
O8—H8	0.8200	C14—C19	1.522 (2)
N1—C1	1.322 (2)	C15—C13^ii^	1.400 (2)
N1—C12	1.363 (2)	C15—C20	1.493 (2)
N2—C10	1.318 (2)	C16—C18^iii^	1.386 (2)
N2—C11	1.364 (2)	C16—C17	1.404 (2)
C1—C2	1.399 (3)	C16—C21	1.522 (2)
C1—H1A	0.9300	C17—C18	1.393 (2)
C2—C3	1.372 (3)	C17—C22	1.490 (2)
C2—H2A	0.9300	C18—C16^iii^	1.386 (2)
C3—C4	1.395 (3)	C18—H16A	0.9300
C3—H3A	0.9300		
			
O1W—Mn1—O5^i^	85.93 (5)	C8—C7—C6	123.54 (17)
O1W—Mn1—O7	99.43 (6)	C11—C7—C6	118.32 (19)
O5^i^—Mn1—O7	96.43 (5)	C9—C8—C7	119.84 (17)
O1W—Mn1—O1	86.66 (6)	C9—C8—H8A	120.1
O5^i^—Mn1—O1	172.36 (4)	C7—C8—H8A	120.1
O7—Mn1—O1	82.92 (4)	C8—C9—C10	118.7 (2)
O1W—Mn1—N2	96.43 (6)	C8—C9—H9A	120.6
O5^i^—Mn1—N2	84.21 (5)	C10—C9—H9A	120.6
O7—Mn1—N2	164.13 (5)	N2—C10—C9	122.82 (18)
O1—Mn1—N2	98.51 (5)	N2—C10—H10A	118.6
O1W—Mn1—N1	171.56 (5)	C9—C10—H10A	118.6
O5^i^—Mn1—N1	91.39 (5)	N2—C11—C7	121.88 (17)
O7—Mn1—N1	88.81 (6)	N2—C11—C12	118.21 (14)
O1—Mn1—N1	96.21 (5)	C7—C11—C12	119.90 (16)
N2—Mn1—N1	75.32 (6)	N1—C12—C4	121.70 (17)
C19—O1—Mn1	141.94 (10)	N1—C12—C11	118.23 (14)
Mn1—O1W—H1WB	103.6 (17)	C4—C12—C11	120.05 (16)
Mn1—O1W—H1WA	131.1 (18)	C14—C13—C15^ii^	121.62 (14)
H1WB—O1W—H1WA	111 (2)	C14—C13—H13A	119.2
C20—O4—H4	109.5	C15^ii^—C13—H13A	119.2
C21—O5—Mn1^i^	131.34 (10)	C13—C14—C15	118.80 (14)
C22—O7—Mn1	132.25 (10)	C13—C14—C19	117.00 (13)
C22—O8—H8	109.5	C15—C14—C19	124.16 (14)
C1—N1—C12	118.72 (15)	C14—C15—C13^ii^	119.58 (15)
C1—N1—Mn1	127.15 (12)	C14—C15—C20	120.56 (14)
C12—N1—Mn1	114.05 (11)	C13^ii^—C15—C20	119.85 (14)
C10—N2—C11	118.60 (15)	C18^iii^—C16—C17	118.29 (14)
C10—N2—Mn1	126.98 (11)	C18^iii^—C16—C21	116.70 (12)
C11—N2—Mn1	113.96 (11)	C17—C16—C21	124.95 (14)
N1—C1—C2	123.36 (18)	C18—C17—C16	119.83 (14)
N1—C1—H1A	118.3	C18—C17—C22	119.82 (13)
C2—C1—H1A	118.3	C16—C17—C22	120.31 (14)
C3—C2—C1	118.06 (19)	C16^iii^—C18—C17	121.88 (13)
C3—C2—H2A	121.0	C16^iii^—C18—H16A	119.1
C1—C2—H2A	121.0	C17—C18—H16A	119.1
C2—C3—C4	120.50 (17)	O2—C19—O1	124.21 (14)
C2—C3—H3A	119.8	O2—C19—C14	119.47 (13)
C4—C3—H3A	119.8	O1—C19—C14	116.15 (13)
C3—C4—C12	117.66 (17)	O3—C20—O4	123.94 (16)
C3—C4—C5	123.69 (17)	O3—C20—C15	123.99 (15)
C12—C4—C5	118.61 (19)	O4—C20—C15	112.06 (13)
C6—C5—C4	121.33 (18)	O5—C21—O6	125.93 (14)
C6—C5—H5A	119.3	O5—C21—C16	116.50 (13)
C4—C5—H5A	119.3	O6—C21—C16	117.22 (14)
C5—C6—C7	121.77 (17)	O7—C22—O8	124.29 (14)
C5—C6—H6A	119.1	O7—C22—C17	120.20 (13)
C7—C6—H6A	119.1	O8—C22—C17	115.50 (14)
C8—C7—C11	118.12 (17)		
			
O1W—Mn1—O1—C19	−130.08 (17)	C6—C7—C11—N2	−179.99 (15)
O7—Mn1—O1—C19	129.98 (17)	C8—C7—C11—C12	177.39 (15)
N2—Mn1—O1—C19	−34.06 (17)	C6—C7—C11—C12	−0.9 (2)
N1—Mn1—O1—C19	41.96 (17)	C1—N1—C12—C4	0.2 (2)
O1W—Mn1—O7—C22	−69.40 (15)	Mn1—N1—C12—C4	−176.96 (12)
O5^i^—Mn1—O7—C22	−156.33 (14)	C1—N1—C12—C11	178.22 (14)
O1—Mn1—O7—C22	16.00 (14)	Mn1—N1—C12—C11	1.03 (18)
N2—Mn1—O7—C22	112.2 (2)	C3—C4—C12—N1	−1.0 (2)
N1—Mn1—O7—C22	112.41 (15)	C5—C4—C12—N1	176.62 (15)
O5^i^—Mn1—N1—C1	−95.99 (14)	C3—C4—C12—C11	−178.99 (15)
O7—Mn1—N1—C1	0.42 (14)	C5—C4—C12—C11	−1.3 (2)
O1—Mn1—N1—C1	83.16 (14)	N2—C11—C12—N1	2.8 (2)
N2—Mn1—N1—C1	−179.63 (15)	C7—C11—C12—N1	−176.29 (14)
O5^i^—Mn1—N1—C12	80.91 (11)	N2—C11—C12—C4	−179.13 (14)
O7—Mn1—N1—C12	177.32 (11)	C7—C11—C12—C4	1.7 (2)
O1—Mn1—N1—C12	−99.93 (11)	C15^ii^—C13—C14—C15	0.1 (2)
N2—Mn1—N1—C12	−2.73 (10)	C15^ii^—C13—C14—C19	177.97 (13)
O1W—Mn1—N2—C10	−2.02 (15)	C13—C14—C15—C13^ii^	−0.1 (2)
O5^i^—Mn1—N2—C10	83.21 (14)	C19—C14—C15—C13^ii^	−177.80 (13)
O7—Mn1—N2—C10	176.37 (15)	C13—C14—C15—C20	−179.72 (13)
O1—Mn1—N2—C10	−89.58 (15)	C19—C14—C15—C20	2.6 (2)
N1—Mn1—N2—C10	176.18 (15)	C18^iii^—C16—C17—C18	0.1 (2)
O1W—Mn1—N2—C11	−174.03 (11)	C21—C16—C17—C18	177.34 (13)
O5^i^—Mn1—N2—C11	−88.80 (11)	C18^iii^—C16—C17—C22	177.73 (13)
O7—Mn1—N2—C11	4.4 (2)	C21—C16—C17—C22	−5.0 (2)
O1—Mn1—N2—C11	98.40 (11)	C16—C17—C18—C16^iii^	−0.1 (2)
N1—Mn1—N2—C11	4.17 (10)	C22—C17—C18—C16^iii^	−177.74 (14)
C12—N1—C1—C2	0.6 (3)	Mn1—O1—C19—O2	−168.79 (11)
Mn1—N1—C1—C2	177.34 (13)	Mn1—O1—C19—C14	16.0 (2)
N1—C1—C2—C3	−0.5 (3)	C13—C14—C19—O2	−93.97 (19)
C1—C2—C3—C4	−0.4 (3)	C15—C14—C19—O2	83.76 (19)
C2—C3—C4—C12	1.1 (3)	C13—C14—C19—O1	81.52 (17)
C2—C3—C4—C5	−176.44 (17)	C15—C14—C19—O1	−100.75 (18)
C3—C4—C5—C6	177.60 (18)	C14—C15—C20—O3	6.7 (2)
C12—C4—C5—C6	0.1 (3)	C13^ii^—C15—C20—O3	−172.92 (15)
C4—C5—C6—C7	0.8 (3)	C14—C15—C20—O4	−173.91 (14)
C5—C6—C7—C8	−178.54 (18)	C13^ii^—C15—C20—O4	6.5 (2)
C5—C6—C7—C11	−0.4 (3)	Mn1^i^—O5—C21—O6	31.1 (2)
C11—C7—C8—C9	0.8 (3)	Mn1^i^—O5—C21—C16	−141.87 (11)
C6—C7—C8—C9	178.96 (17)	C18^iii^—C16—C21—O5	93.87 (17)
C7—C8—C9—C10	0.6 (3)	C17—C16—C21—O5	−83.45 (18)
C11—N2—C10—C9	0.2 (3)	C18^iii^—C16—C21—O6	−79.70 (17)
Mn1—N2—C10—C9	−171.46 (13)	C17—C16—C21—O6	102.98 (18)
C8—C9—C10—N2	−1.1 (3)	Mn1—O7—C22—O8	0.8 (2)
C10—N2—C11—C7	1.2 (2)	Mn1—O7—C22—C17	−178.26 (10)
Mn1—N2—C11—C7	173.95 (12)	C18—C17—C22—O7	177.64 (14)
C10—N2—C11—C12	−177.90 (15)	C16—C17—C22—O7	0.0 (2)
Mn1—N2—C11—C12	−5.16 (18)	C18—C17—C22—O8	−1.5 (2)
C8—C7—C11—N2	−1.7 (2)	C16—C17—C22—O8	−179.20 (14)

Symmetry codes: (i) −*x*, −*y*, −*z*+2; (ii) −*x*, −*y*+1, −*z*+1; (iii) −*x*−1, −*y*, −*z*+2.

### Hydrogen-bond geometry (Å, º)

**Table:**

*D*—H···*A*	*D*—H	H···*A*	*D*···*A*	*D*—H···*A*
O8—H8···O1	0.82	1.75	2.5176 (16)	155
O4—H4···O6^iv^	0.82	1.81	2.592 (2)	158
O1*W*—H1*WB*···O6^i^	0.79 (2)	1.95 (3)	2.7108 (19)	160 (2)
O1*W*—H1*WA*···O2^v^	0.80 (2)	1.94 (2)	2.7117 (18)	162 (2)

Symmetry codes: (i) −*x*, −*y*, −*z*+2; (iv) −*x*−1, −*y*+1, −*z*+2; (v) −*x*, −*y*, −*z*+1.
